# Octacalcium phosphate/gelatin composite facilitates bone regeneration of critical-sized mandibular defects in rats: A quantitative study

**DOI:** 10.15171/joddd.2019.040

**Published:** 2019

**Authors:** Fereydoon Sargolzaei-Aval, Eshagh Ali Saberi, Mohammad Reza Arab, Narjes Sargolzaei, Tayebeh Sanchooli, Sima Tavakolinezhad

**Affiliations:** ^1^Cellular and Molecular Research Center and Department of Anatomical Sciences, School of Medicine, Zahedan University of Medical Sciences, Zahedan, Iran; ^2^Department of Endodontics, School of Dentistry, Zahedan University of Medical Sciences, Zahedan, Iran; ^3^Department of Community Medicine, School of Medicine, Zahedan University of Medical Sciences, Zahedan, Iran

**Keywords:** Dental enamel, fluorides, hardness

## Abstract

***Background.*** Regeneration of bone defects remains a challenge for maxillofacial surgeons. The present study aimed to compare the effects of octacalcium phosphate (OCP) and the combination of octacalcium phosphate/gelatin (OCP/Gel) on mandibular bone regeneration in rats

***Methods.*** In the present study, 36 male Sprague-Dawley rats were used. The animals were randomly assigned to the following experimental groups: OCP (n=12), OCP/Gel (n=12), and the control group (n=12). Defects were created in the rat mandibles and filled with 10 mg of OCP and OCP/Gel disks in the experimental groups. In the control group, however, no substance was administered. Samples were taken on days 7, 14, 21 and 56, respectively, after the implantation. Sections (5 µ) were prepared and stained by H&E. The sections were studied, and the volume fraction of newly formed bone was measured by Dunnett's T3 test based on the significance level (P=0.05).

***Results.*** In the experimental groups, the new bone formation began from the margin of defects 7‒14 days after the implantation. During the healing process, the newly formed bone healed a larger area of the defects and grew structurally. In the control group, the defects were primarily filled with dense connective tissue, and only a small amount of new bone was formed. The present study showed a statistically significant difference in the volume of newly formed bone between the experimental groups and the control group (P<0.001).

***Conclusion.*** OCP/Gel composite can be beneficial in the healing process of mandibular bone defects.

## Introduction

Regeneration of bone defects caused by trauma, infection or tumor resection is a significant challenge in maxillofacial and reparative surgeries. Although a small number of defects are successfully managed by autogenous bone grafts, a large number of defects require biocompatible bone graft substitutes.^1^ Several types of synthetic bone substitutes are available. These materials are either osteoinductive or osteoconductive and serve as a carrier for the maintenance and gradual release of bone morphogenetic proteins (BMPs).^2-5^

Calcium phosphate derivatives are one of the most frequently applied synthetic biomaterials that induce the regeneration of hard tissues.^6^ These materials are divided into different subtypes, depending on the ratio of calcium and phosphate in their composition. They resemble the natural mineral content of bone and teeth in terms of physical and chemical properties.^7,8^ Different compositions of calcium phosphate have variable potentials for inducing new bone formation and repairing bone defects. Some of them serve as carriers for BMPs,^9,10^ while others are osteoinductive.^11,12^ Octacalcium phosphate, Ca_8_H_2_(PO_4_)_6_ 5H_2_O, is the most soluble salt; thus, the use of synthetic OCP was considered as a potential factor for the nucleation of bone induction in orthotopic sites, which could be replaced by a significantly higher volume of newly formed bone rather than the other calcium phosphate phases, such as HA^13,14^ or amorphous carbonated apatite.^15^ The biodegradable characteristics of OCP are acquired via its resorption by osteoclast-like multinucleated giant cells (MNGCs) in bone marrow spaces.^11,16^

On the other hand, in animal models (rodents and canines), it has been reported that the bone formation in large bone defects in maxillofacial regions is regulated by the granule size of calcium phosphate materials.^17^ Distinct granule size might provide distinct inter-granular spaces, considering the involvement of mechanisms the same as the porous structure, encouraging cell migration in a three-dimensional scaffold and leading to better bone formation.^18^

Although OCP possesses many desirable properties as a bone substitute, it cannot be molded using sintering processes due to its intrinsic crystal structure. In order to resolve the disadvantages and improve the handling property, a combination of OCP and gelatin (OCP/Gel) was prepared.^19^ Thus, the majority of OCP-based materials were formed as an implantable scaffold with a better handling property in clinical situations.^9^ Combining OCP with natural polymers improved both the handling property and the osteoconductivity.^20^ Based on previous studies, collagen and gelatin increase the biological properties of OCP in bone regeneration.^21,22^

Gelatin, which is a natural material derived from collagen with the same composition, has been widely used as a matrix to obtain biomimetic calcium phosphate (CaP) in bone regeneration and tissue engineering fields.^23,24^ In addition, gelatin has been increasingly used as the organic moiety in polymer‒inorganic hybrids due to its biodegradability, biocompatibility, hydrogel characteristics, availability and cost-effectiveness.^25^

In an experimental study, Saito et al^26^ evaluated the efficacy of OCP/Gel composites in bone regeneration in a rabbit tibial defect model and suggested that the composites had a porous structure of up to 500 μm in diameter and maintained the OCP structure regardless of its content in a gelatin matrix. The OCP/Gel composite with a higher OCP content showed greater bone regeneration and tended to undergo faster biodegradation in both cortical bone and bone marrow regions. These results suggest that the biodegradation tendency of the composite could be accelerated by increasing the OCP content.

The present study aimed to investigate the effects of octacalcium phosphate and octacalcium phosphate/gelatin composite on the repair of rat mandibular bone defects.

## Methods

### Preparation of materials

Gelatin was purchased from Sigma (Type B, Sigma-Aldrich Co. Louis MO, USA), and synthetic OCP [Ca_8_H_2_(PO_4_)6.5H_2_O] was prepared by mixing a calcium and phosphate solution as described previously.^27,28^ The granules consisting of an OCP crystal aggregate were prepared by lightly grinding the dried OCP cake, using a pestle and mortar and then passing it through a standard testing sieve. Granules with diameters in the range of 300‒500 μm were used for implantation. The sieved granules were sterilized by heating at 120ºC for two hours in an oven before implantation. A previous study showed that such heating does not affect the physical properties, such as the crystalline structure or specific surface area of OCP granules.^29^ Synthetic OCP/Gel composite was prepared following the mentioned methods.^19^ Briefly, gelatin was dissolved in distilled water. OCP granules were added to the 3.6% gelatin (Gel) solution and mixed. Then, the OCP/Gel composite containing 64 wt% (OCP 64/Gel) was lyophilized, and the block was molded (5 mm in diameter, 2 mm in thickness). The molded OCP/Gel composite underwent dehydrothermal treatment (150ºC, 24 hours) and was then sterilized.

### Animals and surgical procedures

This experimental study was conducted on 36 adults (6‒8 weeks old) male Sprague-Dawley rats with a mean weight of 120‒150 g. The animals were randomly allocated to two experimental (OCP and OCP/Gel) groups and one control group and kept under standard conditions with light/dark cycles of equal duration. The guideline and regulations for laboratory animal care and welfare and national law were followed. All the procedures were approved by the Ethics Committee for Animal Experiments of Zahedan University of Medical Sciences (IR.ZAUMS.REC.1395.87).

The animals were anesthetized by intraperitoneal injection of 60 mg/kg of ketamine hydrochloride (Ketalar, Trustech Pharma-Care, Bayern, Germany) and 20 mg/mL of xylazine (Pantex Holland B.V., Duizel, Netherland) at 2:1 ratio. Diethyl ether was used for maintenance of anesthesia. After the induction of general anesthesia, the animals were fixed on the operating table in a supine position. The respective area on the body of the mandible was shaved and disinfected using 10% Betadine (Toliddarou, Tehran, Iran). Both sides of the mandible were incised by 1.5 cm using a sterile surgical scalpel, and a full-thickness periosteal flap was elevated. A critical-sized trephine defect, measuring 5×2 mm (diameter and depth),^30^ was drilled in the body of the mandible close to the alveolar crest, using a dental drill and irrigated with a cold saline solution. In the experimental groups, a block of OCP/Gel (OCP/Gel group) and granules of OCP (OCP group), equal to the volume of the created defect (10 mg, previously prepared and packed), were then implanted into the trephine defect, respectively. In the control group, the animals were processed in the same way as the experimental group except for the implantation after defect creation. In the next step, the skin and the underlying connective tissue at the surgical site were sutured in two layers, using a 4/0 absorbable chromic suture in the same way (Catgut, Wei Gao Group Kanglida Medical Products Co, LTD. Heze, China). To prevent infection, gentamicin (20 mg/kg) (Exir Company, Iran) was injected intramuscularly for three days after the surgery.

### Tissue preparation

In all the experimental and control groups, tissue samples were harvested on the 7th, 14th, 21st and 56th days (from six rats sacrificed at each time interval) to prepare histologic sections. At the mentioned time intervals, general anesthesia was induced by intraperitoneal injection of ketamine hydrochloride. The chest was opened, and 10% buffered formalin was perfused through the heart to achieve in situ fixation of tissues. The respective area with a margin of host bone was resected and stored in 10% buffered formalin at room temperature for one week for better fixation. Then, the tissue specimens were decalcified through immersion in a decalcifying solution containing 10% formic acid, 2.9% citric acid and 1.8% trisodium citrate dihydrate at room temperature for four weeks.^31^ Following the preparation of paraffin blocks, 5-micron sections were serially obtained for histologic and histomorphometric examinations. They were stained with hematoxylin-eosin (H&E). Sections obtained from all the three groups on the 7th, 14th and 21st days of post-implantation were histologically assessed using light microscopy (Zeiss, Carl Zeiss Microscopy GmbH. Goettingen, Germany), and those obtained on the 56th post-implanted day were assessed both histologically and histomorphometrically.

### Histologic evaluation

Histologic analysis of the specimens was carried out blindly under a light microscope by a histologist blinded to the type of bone substitutes used. Photomicrographs were taken using a photomicroscope (Leica DM 500, Leica Microsystems, GmbH. Wetzler, Germany) on the 7th, 14th, and 21st days of the experiment.

### Histomorphometric assessment

Six sections obtained on the 56th day (from each group) were randomly selected: (two sections from the surface, two sections from the middle section, and two sections from the deep section of the created defect) and placed on three slides (two sections on each slide) for assessing histomorphometric and determining the volume of the newly formed bone in all the groups. Ultimately, 18 slides (36 sections) were selected for histomorphometric evaluation in each group. The sections were stained by H&E and evaluated under a light microscope equipped with an eyepiece graticule at ×40 magnification based on the point-counting technique. The mean volume of newly formed bone was calculated in all the groups.^32^

### Statistical analysis

Histomorphometric data were analyzed using SPSS 20. Means, SDs, modes and medians were calculated. Dunnett's T3 test was used to compare the means in all the groups. P<0.05 was considered as statistically significant.

## Results

### Histologic assessments

**Figure 1:**
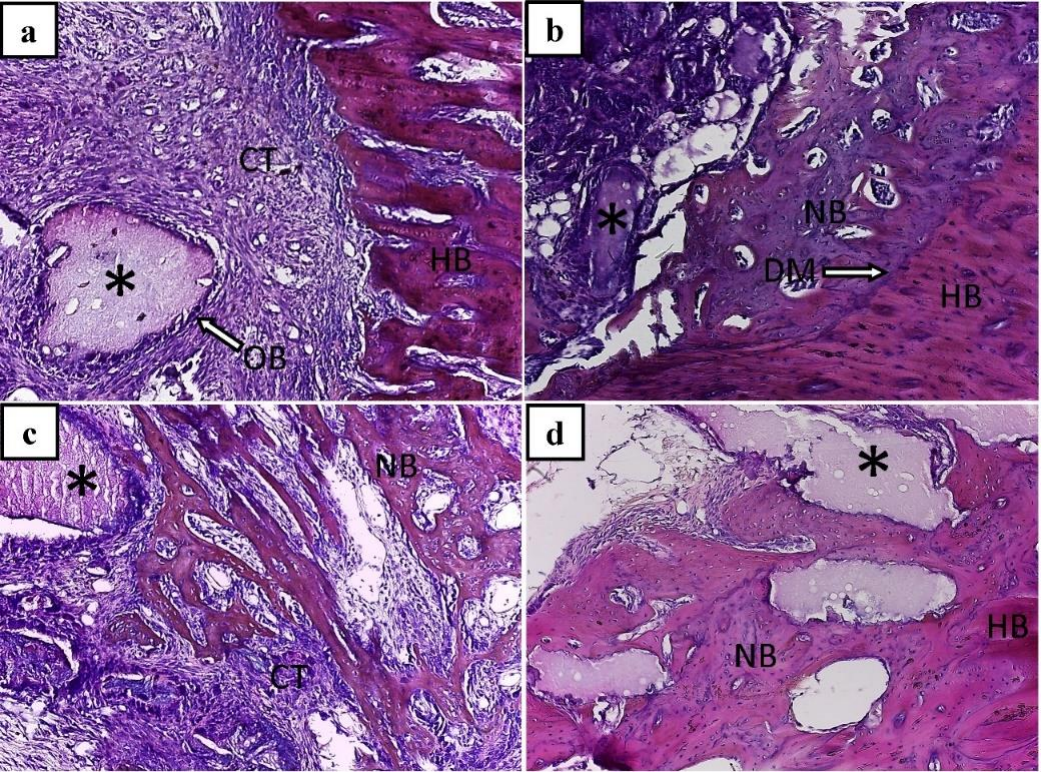
Histologic micrograph of the OCP group at 7-day (a), 14-day (b) 21-day (c) and 56-day (d) post-implantation intervals. OCP particles (*), connective tissue (CT), host bone (HB), newly formed bone (NB), defect margin (DM), osteoblast cell (OB). H&E staining, magnification = a, b, c, d (×10).

In the OCP experimental group, the ability of OCP implanted particles in the differentiation of connective tissue cells and adherence of these cells to the implanted particles was one of the most important findings of this experimental group on the 7th day after implantation. The cuboidal shape of the cells adhering to the margins of the implanted particles and their basophilia suggested the differentiation of the first osteoblastic-like cells in this group during this period (Figure 1a). On the 14th day after implantation, the OCP implanted particles had a greater ability to induce bone formation (Figure 1b). On the 21st day after implantation, the first Haversian systems appeared with vascular connective tissue between newly formed bones. In addition, during this period, active absorption of the implanted particles by multi-nuclear pre-osteoclast cells was observed adjacent to the implanted particles (Figure 1c). On the final day of the study, the newly formed bone with a remarkable increase in volume exhibited a high degree of tissue organization (Figure 1d).

**Figure 2:**
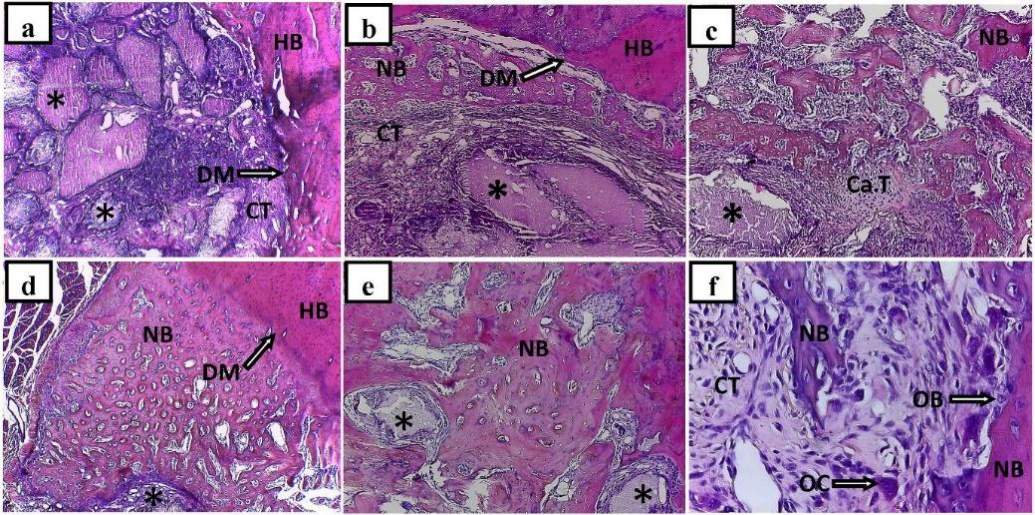
Histologic micrograph of the OCP/G group at 7-day (a), 14-day (b), 21-day (c,d) and 56-day (e,f) post-implantation intervals. Implanted materials (OCP particle) (*) CT: connective tissue. DM: defect margin. HB: host bone. NB: newly formed bone. CaT: cartilaginous tissue. OC: osteoclast cells. OB: osteoblast cells. H&E staining, magnification = a, d (×4), b, c, e (×10), f (×40).

In the OCP/Gel experimental group, the defect area was mainly filled by young and hypercellular connective tissue and gelatin on the 7th day after the implantation and OCP implanted materials characterized by clear basophilic and eosinophilic properties were observed. During this period, an implanted material-induced cellular response was seen; however, there were no detectable signs of osteogenesis (Figure 2a). On the 14th day after implantation, implanted material-induced osteogenesis was detectable near the defect margin and the host bone. The eosinophilic osteoid that featured the first signs of the extracellular matrix organization was well detectable. The signs of the inducing osteoblast-like cells were evident in some parts of the newly formed bone, adjacent to the implanted material, indicating the inductive effects of the implanted materials on the young connective tissue surrounding these materials and the newly formed bone surface (Figure 2b). On the 21st day after the implantation, a new bone tissue generated in the form of primary osteons with areas of the woven bone and islands of cartilaginous tissue were observed around the defect areas and the implanted materials. On the newly formed bone islands, a dense connective tissue was observed, which indicated a high tendency of implanted materials to induce new bone formation through both conventional methods of bone formation (Figure 2c). In other specimens at this time interval, the signs of bone maturation with the Haversian systems and its components were clearly visible (Figure 2d). On the final day of the study, the signs of new bone maturation were observed, and some parts of the implanted materials which had not been absorbed were visible (Figure 2e). In other specimens at this time interval, the signs of cellular differentiation and formation of eosinophilic multinucleated osteoclast cells along with a dense connective tissue around the emerging bone spicules were observed. In addition, on the surface of these emerging bone spicules, the osteoblast-like cells, which showed a specific epithelioid appearance, were also visible (Figure 2f).

**Figure 3:**
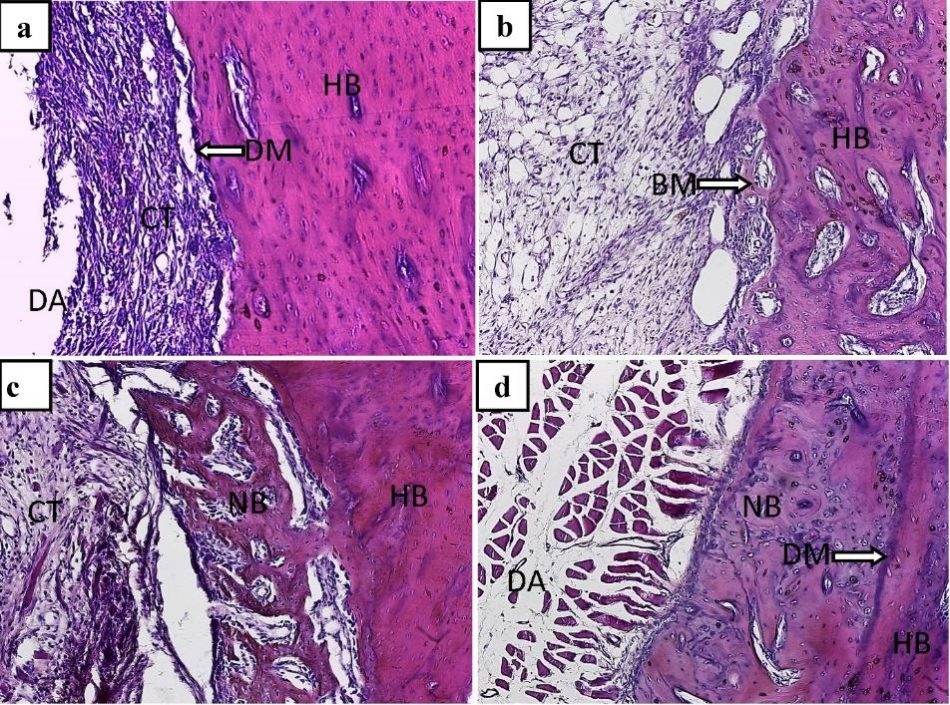
Histologic micrograph of the control group at 7-day (a), 14-day (b) 21-day (c) and 56-day (d) post-implantation intervals. Connective tissue (CT), host bone (HB), newly formed bone (NB), defect margin (DM), defect area (DA). H&E staining, magnification = a, b, c, d (×10).

In the control group, the defect area was filled with young connective tissue with a regular and completely defined host bone margin on the 7th day after surgery (Figure 3a). On the 14th day, the connective tissue filling the defect area adjacent to the defect margin had a greater organization than the deep parts and the first signs of bone tissue formation, appearing as a cluster of newly formed bone matrix near the margin of the bone defects (Figure 3b). On the 21st day, the volume of the newly formed bone increased, and the new bone tissue organization adjacent to the host bone was greater than the distant parts (Figure 3c). On the final day of the study, the new bone tissue that enhanced full organization was formed. The strong attachment of this newly formed bone tissue to the host bone is one of the most important findings of this period (Figure 3d).

### Histomorphometric results

**Figure 4:**
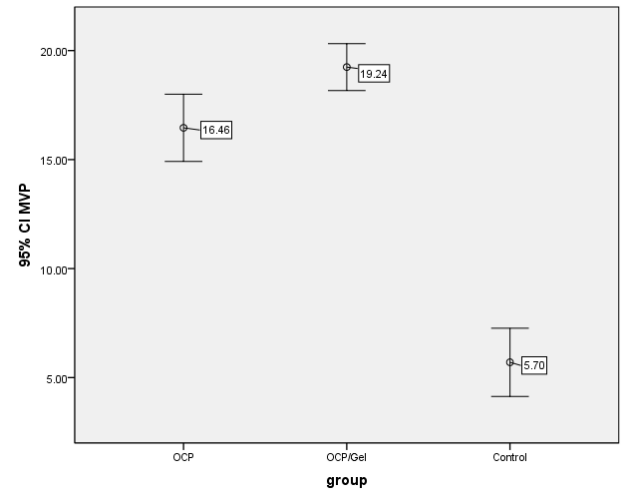
Comparison of the mean volume percentage (MVP) of newly formed bone between the experimental and control groups.

The volume of newly formed bone in the experimental and control groups on the 56th day after surgery and implanted materials was calculated using the point-counting technique, and the mean value was reported as a volume fraction up to two decimals for each group (Table 1 and Figure 4). Since the volume of newly formed bone between the experimental and control groups did not have equal variances, Dunnett's T3 test was used. The highest mean volume of newly formed bone was observed in the OCP/Gel and OCP experimental groups. The control group had the lowest volume of newly formed bone and statistically significant differences from both experimental groups (P<0.001), which indicated the positive effect of implanting materials on healing bone defects in the mandibular bone. There was a statistically significant difference between the experimental OCP/Gel and OCP groups, with the highest volume of newly formed bone (P<0.011).

**Table 1:** Comparison of the volume percent of newly formed bone among the experimental and control groups [OCP (octacalcium phosphate), Gel (gelatin), OCP/Gel (octacalcium phosphate/gelatin)]

**Groups**		**Number of microscopic fields**	**Mean**	**Standard deviation**	**Median**	**Mode (percentage )**	**Min**	**Max**	**P- value**
**OCP**	OCP/Gel	144	19.24	6.53	21	25 (31.9)	0	25	0.011
	Control	144	5.70	9.49	0	0 (65.3)	0	25	<0.001
**OC P/Gel**	OCP	144	16.45	9.35	21	25 (37.5)	0	25	0.011
	Control	144	5.70	9.49	0	0 (65.3)	0	25	<0.001
**Control**	OCP	144	16.45	9.35	21	25 (37.5)	0	25	<0.001
	OCP/Gel	144	19.24	6.53	21	25 (31.9)	0	25	<0.001

## Discussion

The efficacy of synthetic materials alone or in combination with one another for the regeneration of bone defects has been the topic of previous studies.^3-8^ The present study aimed to assess the osteoinductive potential of OCP alone and in combination with gelatin in bone regeneration after being implanted in mandibular bone defects of rats. The effect of these materials on developing the process of bone formation was studied qualitatively and quantitatively using light microscopy. The histomorphometric and bone tissue volume were analyzed only for day 56 to compare the experimental groups with the control group.

The results of the present study in the OCP experimental group showed that OCP induced intramembranous bone formation after being implanted in the defect area in the mandibular bone. This indicated a host response to this material. Kamakura et al^33^ reported a similar reaction to OCP implantation at the site of defects created in the parietal bone. On the 21st day after the implantation, bone trabecula with an irregular pattern was seen at the defect site in the OCP group in our study. Osteoblasts were noted on the surface, and osteocytes were clearly seen at the center of bone trabeculae, indicating the organization of the newly formed bone trabeculae and their remodeling. Most researchers found similar results in the fourth week after the implantation of OCP particles.^28,34^ The inconsistency between the results of this study and those of the present one could be attributed to the role of the implanted material in the deeper sections of the defect. Finally, the results of the present study suggested that the first step for osteogenesis in this experimental group was induced by the host bone at the most peripheral areas of the defect and then gradually extended towards the center.

Although the functional relationship between osteoblasts forming a new bone matrix on OCP and osteoclasts resorbing OCP has not yet been fully elucidated, the enhancement of bone formation and OCP biodegradation (followed by a remodeling-like mechanism by osteoblasts and osteoclasts in normal bone tissue) has been reported in various bone defects when OCP particles are implanted in the medullary canal of rabbit femur^11^ and the subperiosteal region of mouse calvaria.^30^ Based on these results, it seems likely that the enhancement of bone formation by OCP is accompanied by simultaneous activation of osteoclastic resorption of OCP.^17^ On the other hand, the mechanism to enhance these cellular activities was originated from the intrinsic chemical property of OCP, which can be progressively converted to Ca-deficient hydroxyapatite under physiological conditions. Further, it provides a suitable surface for cell attachment and differentiation through inorganic ionic dissolution and protein interactions between the tissue fluid and the hydrolyzing surface. Thus, OCP could have a stimulatory capacity on the calcified tissue cells that could break the cells apart from the materials.^35^

In the present study, in the OCP/Gel group, connective tissue fibers, and the first signs of osteoblastic differentiation were noted on the 7th and 14th days following implantation. This finding is consistent with those of some other studies (Suzuki et al and Handa et al).^36,19^ On the 21st day, in addition to the woven bone formed at the defect margins, cartilaginous tissue masses were observed at the center of the defects; this phenomenon has not been reported in any of the previous studies. The induction of cells and their mechanisms of action in response to the implanted materials are unclear, but it can be influenced by the simultaneous interaction between the host’s bone and the implanted materials. Another important finding in this group was the penetration of the connective tissue into the implanted materials that induced the differentiation of osteoblast cells and eventually, the formation of new bone mass among the implanted particles in deep parts of the defect. Suzuki et al^36^ reported that this phenomenon is caused by stimulatory effect of OCP on the induction of osteoblasts and osteoclasts, which can migrate into and through the implanted materials with the host’s osteoblasts and cause bone formation in the central parts of the defect, which is consistent with the results of the present study.

Finally, a relatively complete bone formation was observed in all areas of the defect, and the implanted material was completely absorbed. This absorption rate is in contrast with the study of Handa et al.^19^ The inconsistency between the results of his study and those of the present study may be due to the differences in the amount of gelatin used in OCP/Gel composite. In the present study, 64% of octacalcium phosphate and in the study of Handa et al, 40% of it was used, leading to the slow release of octacalcium phosphate particles in the composite and maintaining up to sixteen weeks after implanting the materials. On the other hand, according to Ishiko-Uzuka’s study,^37^ the gelatin matrix might support the OCP efficacy to induce new bone formation with better quality. The capability of OCP to stimulate the osteoclastic formation and the differentiation of the osteoblasts might be involved in activating bone metabolism around the implanted site.^37^ Thus, it is possible that gelatin molecules present in the compound used in the present study influenced these bone-related cells.

Considering that OCP possesses both osteoinductive and osteoconductive properties,^29,38^ and gelatin serves as a carrier and scaffold,^21^ it seems that the interaction of these two materials induces bone formation by promoting direct differentiation of undifferentiated mesenchymal cells to osteoblasts. Further, the interaction effect of these two materials might even result in the differentiation of some of the mesenchymal cells to chondroblasts or chondroblast-like cells observed between the particles and at the defect margins. This new finding has not yet been reported in any previous study.

Based on the histomorphometric results, the mean volume of newly formed bone was significantly different between the experimental groups and control group (P<0.001), indicating the positive effects of implanted materials on the defect, consistent with the results of the study of Suzuki et al.^31^ It was demonstrated that the highest volume of newly formed bone was related to OCP/Gel experimental groups (19.24) and then OCP experimental group (16.45) with a noticeable difference. The volume of newly formed bone was statistically different between the two experimental groups (P<0.011); this is probably due to the increased weight of OCP combined with gelatin in the OCP/Gel composite group.

The effect of the gelatin matrix with OCP might have also accelerated the biodegradation of the OCP particles.^39^ Studies showed that highly resorbable and osteoconductive biomaterials, such as OCP/Gel composite, have potential use in clinical practice, which is consistent with those of the present study. Further studies are necessary to investigate the relationship between the rapid biodegradability and bone regenerative capacity of the OCP/Gel composite. Besides, similar studies must be conducted using additional evaluations in order to provide the necessary tools to compare materials qualitatively and quantitatively.

## Conclusion

The present study demonstrated that an OCP/Gel composite containing a higher content of OCP in a granule form is able to improve the process of bone formation and defect healing. It suggested that the OCP/Gel composite not only induced intramembranous ossification (which is among the properties of OCP) but also resulted in the induction of endochondral ossification. Interestingly, this finding can lead us toward new treatment strategies for more efficient management of bone defects in maxillofacial and reparative surgeries.

## Acknowledgments

The authors appreciate the personnel of the Animal Research Institute at Zahedan University of Medical Sciences.

## Competing Interests

The authors deny any conflict of interest related to this study.

## Authors’ Contributions

FSA and MRA designed the study. FSA, EAS and TS performed the experiments. FSA, MRA and NS collected data and carried out statistical analyses. FSA and MRA prepared the manuscript. FSA and ST revised the manuscript.

## Funding

Not applicable.

## Ethics Approval

The guideline and regulations for laboratory animal care and welfare, as well as national law, were followed. All the procedures were approved by the Ethics Committee for Animal Experiments of Zahedan University of Medical Sciences (IR.ZAUMS.REC.1395.87).
